# Theoretical Study on Synchronous Characterization of Surface and Interfacial Mechanical Properties of Thin-Film/Substrate Systems with Residual Stress Based on Pressure Blister Test Technique

**DOI:** 10.3390/polym10010049

**Published:** 2018-01-07

**Authors:** Zhi-xin Yang, Jun-yi Sun, Ke Li, Yong-sheng Lian, Xiao-ting He, Zhou-lian Zheng

**Affiliations:** 1 School of Civil Engineering, Chongqing University, Chongqing 400045, China; yangzhixin123@126.com (Z.Y.); likecqu@163.com (K.L.); yongsheng-lian@cqu.edu.cn (Y.L.); hexiaoting@cqu.edu.cn (X.H.); zhengzl@cqu.edu.cn (Z.Z.); 2 Key Laboratory of New Technology for Construction of Cities in Mountain Area (Chongqing University), Ministry of Education, Chongqing 400045, China

**Keywords:** thin-film/substrate system, residual stress, pressure blister test, interfacial adhesion energy, synchronous characterization

## Abstract

In this study, based on the pressure blister test technique, a theoretical study on the synchronous characterization of surface and interfacial mechanical properties of thin-film/substrate systems with residual stress was presented, where the problem of axisymmetric deformation of a blistering film with initial stress was analytically solved and its closed-form solution was presented. The expressions to determine Poisson’s ratios, Young’s modulus, and residual stress of surface thin films were derived; the work done by the applied external load and the elastic energy stored in the blistering thin film were analyzed in detail and their expressions were derived; and the interfacial adhesion energy released per unit delamination area of thin-film/substrate (i.e., energy release rate) was finally presented. The synchronous characterization technique presented here has theoretically made a big step forward, due to the consideration for the residual stress in surface thin films.

## 1. Introduction

Thin-film/substrate systems have found increasing application in many advancing technologies, such as mechanics, civil engineering, and biotechnology [1,2,3,4]. The reliability of thin-film/substrate systems depends mainly on the interfacial adhesive strength of thin-film/substrate, which is usually from the polymeric adhesives or the generation of thin films. Therefore, the test results for adhesive strength also reflect the performance of polymeric adhesives or the effect of film generation. Usually, the determination of interfacial adhesive strength by a set of test results needs, at the same time, to know some information such as Poisson’s ratios, Young’s modulus, and residual stress of surface thin films. These information, however, are closely related to the fabrication of thin-film/substrate systems. For example, for the thin films of generation the Poisson’s ratios, Young’s modulus, and residual stress of surface thin films may have difference due to the variations in processing conditions such as temperature, humidity, or the sequence of fabrication procedures, while for the adhesive thin films, the polymeric adhesives may remain on the thin films after delamination (resulting in a change of films before adhesion and after delamination), which will make a change in Poisson’s ratios and Young’s modulus, and the conditions such as temperature and humidity may make a change in residual stress of surface thin films. So, the ideal way is that the interface and surface mechanical properties of thin-film/substrate systems should be determined by a same set of test information. This is the synchronous characterization technique so-called here. However, this technology is still in its infancy [5].

Many test techniques have been used to the mechanical characterization of thin-film/substrate systems, but the mechanical properties of surface and interface are usually characterized separately. Conventional testing methods are mainly as follows: using approaches such as micro tensile, bending beam, ultrasonic wave, resonance frequency, indentation, X ray diffraction, optical fluorescence, Raman spectroscopy, pressure blister (or expansion), and shaft-loading blister to obtain the surface information about Poisson’s ratios, Young’s modulus and residual stress [6,7,8,9,10,11,12,13,14,15]; and using approaches such as vertical drawing, ultracentrifugation, supersonic vibration, transparent tape, peeling, cutting, scraping, X ray, pressure blister (or expansion), and shaft-loading blister to obtain the interface information about adhesive strength of thin-film/substrate [16,17,18,19,20,21,22,23,24,25]. It is obvious that the blister testing method crosses the interface and surface, and consequently it is the best one which can be used to achieve the so-called synchronous characterization technique. Taking into account that the stress concentration is very easy to occur in shaft-loading, in our previous study the pressure blister test technique was used to the interfacial mechanical properties of thin-film/substrate systems [24]. However, our previous study [24] failed to achieve the idea of synchronous characterization of surface and interface, while at the same time, our previous study [5] failed to consider the residual stress in the surface thin-film of a thin-film/substrate system.

If the residual stress is present in the surface thin films, however, the analytical solution to the problem of axisymmetric deformation of a blistering thin film with initial stress will become very complicated, and consequently the concept of synchronous characterization will face some intractable technical difficulties. So, our earlier work [5] is only applicable to the thin-film/substrate systems without residual stress. In fact, the residual stress is very easy to be present in the surface thin films due to the variations in use and processing conditions such as temperature and humidity, etc., including the case where the polymeric adhesive remains on the blistering thin films after delamination (usually the polymeric adhesive may produce contraction or expansion). So, the residual stress should be considered into the synchronous characterization technology. This is also the key issue on which this study will focus.

This paper is organized as follows: in the following section, the axisymmetric deformation problem of a blistering thin film was simplified into a circular membrane problem (a mechanical model) and the closed-form solution of the mechanical model was presented, where the residual stress in the surface thin film of thin-film/substrate systems is taken into account by regarding it as an initial stress of the circular membrane problem, and the so-called small-rotation-angle of membrane was given up. In Section 3, the synchronous characterization of surface and interfacial mechanical properties of thin-film/substrate systems was presented, including the expressions to determine Poisson’s ratios, Young’s modulus, and residual stress of surface thin films, the elastic energy stored in the blistering film, the work done by the applied external load, the elastic strain energy stored in the enclosed compressed air, and the interfacial adhesion energy released per unit delamination area of thin-film/substrate (energy release rate). In Section 4, some important issues were discussed, and Section 5 contains the concluding remarks.

## 2. Membrane Equation and Its Closed-Form Solution

The axisymmetric deformation problem of a blistering thin film with initial stress was simplified into a mechanical model as follows: the problem of axisymmetric deformation of the peripherally fixed circular membrane with initial stress under uniformly distributed transverse loads. The problem of axisymmetric deformation of the peripherally fixed circular membrane without initial stress under uniformly distributed transverse loads was originally dealt with by Hencky, and its power series solution with the small-rotation-angle assumption of membrane was presented [26]. This circular membrane problem is usually called well-known Hencky problem for short. A calculation error in [26] was corrected by Chien [27] and Alekseev [28], respectively. This is the so-called well-known Hencky solution and it is often referred to or cited in a number of related studies [29,30,31,32,33,34]. Sun et al. [35] presented the so-called extended Hencky solution, which is applicable to the membranes with or without initial stress but is still a solution under the small-rotation-angle assumption of membrane. Recently, Sun et al. [36] resolved the well-known Hencky problem and presented a closed-form solution without the small-rotation-angle assumption of membrane but considering no initial stress in membranes. We here deal with the well-known Hencky problem once again, under the condition of giving up the small-rotation-angle assumption of membrane and taking the initial stress into account, in order to meet the requirements of the synchronous characterization. The detail derivation is as follows.

**Figure 1:**
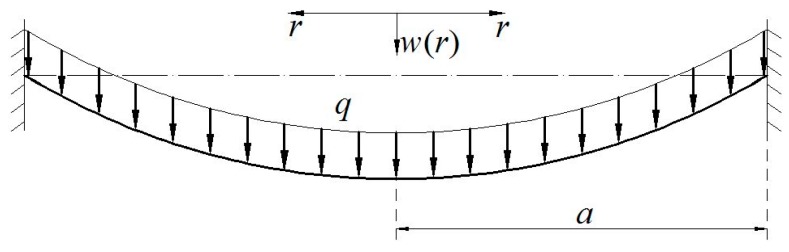
Sketch of the circular membrane problem.

**Figure 2:**
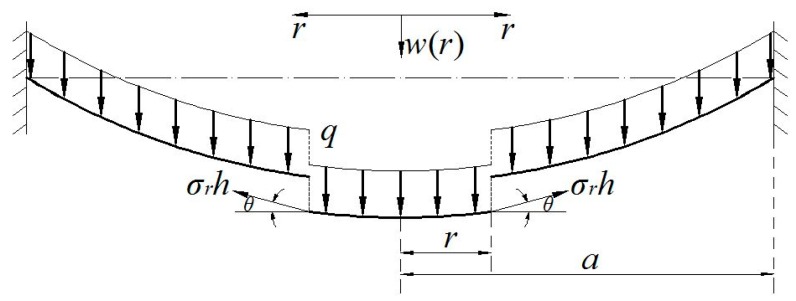
Sketch of the isolated body.

An initially flat elastic circular membrane is fixed at the perimeter of radius a after it is extended a radial plane displacement $u_{0}$ at the radius a, and a uniformly distributed transverse loads q is applied onto the membrane surface, as shown in Figure 1, where r is the radial coordinate and w is the transversal displacement. Suppose that the Young’s modulus of elasticity, Poisson’s ratio, radius, and thickness of the circular membrane are denoted by E, ν, a and h, respectively. An isolated body of radius r (0≤r≤a) is taken from the central portion of the circular membrane to study its static problem of equilibrium, caused by the joint action of the loads q and the membrane force $\sigma_{r}h$ acted on the boundary, as shown just in Figure 2, where $\sigma_{r}$ denotes the radial stress and θ denotes the slope angle of membrane. Here, there are two vertical applied forces, the total force $\pi r^{2}q$ (0≤r≤a) of the loads q and the total vertical force $2\pi rh\sigma_{r}\sin\theta$ produced by the membrane force $\sigma_{r}h$.

The out-plane equilibrium equation is

(1) $$2\pi rh\sigma_{r}\sin\theta =\pi r^{2}q,$$

where

(2) $$\sin\theta =1/\sqrt{1+1/\tan^{2}\theta }=1/\sqrt{1+1/{\left(-dw/dr\right)}^{2}}.$$

Substituting Equation (2) into Equation (1), one has

(3) $$\frac{1}{2}rq\sqrt{1+1/{\left(dw/dr\right)}^{2}}=h\sigma_{r}.$$

The radial and circumferential membrane force in the plane of the circular membrane are $\sigma_{r}h$ and $\sigma_{t}h$, where $\sigma_{t}$ denotes the circumferential membrane stress, then the in-plane equilibrium equation can be written as

(4) $$\frac{d}{dr}(rh\sigma_{r})-h\sigma_{t}=0.$$

For large deflection problems of membrane, the relations of the strain and displacement are

(5a,b) $$e_{r}=\frac{du}{dr}+\frac{1}{2}{\left(\frac{dw}{dr}\right)}^{2},\;e_{t}=\frac{u}{r},$$

where $e_{r}$, $e_{t}$, and u denote the radial strain, circumferential strain, and radial displacement, respectively. The relations of the stress and strain are

(6a,b) $$\sigma_{r}=\frac{E}{1-\nu^{2}}(e_{r}+\nu e_{t}),\;\sigma_{t}=\frac{E}{1-\nu^{2}}(e_{t}+\nu e_{r}).$$

Substituting Equation (5a,b) into Equation (6a,b), it is found that

(7a) $$h\sigma_{r}=\frac{Eh}{1-\nu^{2}}[\frac{du}{dr}+\frac{1}{2}{\left(\frac{dw}{dr}\right)}^{2}+v\frac{u}{r}]$$

and

(7b) $$h\sigma_{t}=\frac{Eh}{1-\nu^{2}}[\frac{u}{r}+\frac{v}{2}{\left(\frac{dw}{dr}\right)}^{2}+v\frac{du}{dr}].$$

From Equations (4), (7a) and (7b), one has

(8) $$\frac{u}{r}=\frac{1}{Eh}(h\sigma_{t}-vh\sigma_{r})=\frac{1}{Eh}[\frac{d}{dr}(rh\sigma_{r})-vh\sigma_{r}].$$

Substitute the u of Equation (8) into Equation (7a), then

(9) $$r\frac{d}{dr}[\frac{1}{r}\frac{d}{dr}(r^{2}h\sigma_{r})]+\frac{Eh}{2}{\left(\frac{dw}{dr}\right)}^{2}=0.$$

It is not necessary to discuss the detailed derivation from Equation (4) to Equation (9), which can be found in any general theory of plates and shells.

The boundary conditions for solving Equations (3), (4), and (9) can be determined, based on the following solution to the problem of plane stretching of the initially flat elastic circular membrane. For the case where the initially flat elastic circular membrane is extended a radial plane displacement $u_{0}$ at r=a, it is obvious that dw(r)/dr=0. So, from Equation (5) it is found that

(10a,b) $$e_{r}=\frac{du}{dr},e_{t}=\frac{u}{r}.$$

Substituting Equation (10a,b) into Equation (6),

(11a,b) $$\sigma_{r}=\frac{E}{1-\nu^{2}}(\frac{du}{dr}+\nu \frac{u}{r}),\sigma_{t}=\frac{E}{1-\nu^{2}}(\frac{u}{r}+\nu \frac{du}{dr}).$$

From Equations (4) and (11a,b), one has

(12) $$r^{2}\frac{d^{2}u}{dr^{2}}+r\frac{du}{dr}-u=0.$$

The boundary conditions for solving Equation (12) are

(13a,b) $$u=0\mathrm{at}r=0,\mathrm{and}u=u_{0}\mathrm{at}r=a.$$

So, under the conditions of Equation (13a,b), the solution of Equation (12) can be written as

(14) $$\frac{u(r)}{r}=\frac{u_{0}}{a}.$$

Substituting Equation (14) into Equations (10a,b) and (11a,b), it is found that

(15a,b) $$e_{r}=e_{t}=\frac{u_{0}}{a}=e_{0},\sigma_{r}=\sigma_{t}=\frac{E}{1-\nu }\frac{u_{0}}{a}=\sigma_{0}.$$

Here, $\sigma_{0}$ denotes the so-called initial stress in the circular membrane after extending a radial plane displacement $u_{0}$ at r=a (the one before applying the uniformly distributed transverse loads q, this initial stress corresponds to the residual stress in the surface thin film of a thin-film–substrate), and $e_{0}$ denotes the initial strain. Equation (15a,b) show that, for the problem of plane stretching of circular membranes, at every point on the stretched circular membrane the radial strain $e_{r}$ is always equal to the circumferential strain $e_{t}$, and also the radial stress $\sigma_{r}$ is always equal to the circumferential stress $\sigma_{t}$. This can be used to explain the reason why the residual stress in the thin film of thin-film/substrate systems is always considered as a uniform stress [37]. To this end, the boundary conditions, under which Equations (3), (4), and (9) can be solved, can finally be written as

(16a,b) $$\frac{u}{r}=\frac{1-\nu }{E}\sigma_{0}\mathrm{and}w=0\mathrm{at}r=a.$$

The following dimensionless variables are introduced,

(17a,b,c,d,e,f) $$Q=\frac{aq}{hE},W=\frac{w}{a},S_{r}=\frac{\sigma_{r}}{E},S_{t}=\frac{\sigma_{t}}{E},S_{0}=\frac{\sigma_{0}}{E},x=\frac{r}{a}.$$

Transform Equations (3), (4), (8), and (9) into

(18) $$x^{2}\frac{d^{2}S_{r}}{dx^{2}}+3x\frac{dS_{r}}{dx}+\frac{1}{2}{\left(\frac{dW}{dx}\right)}^{2}=0,$$

(19) $${\left(\frac{dW}{dx}\right)}^{2}=\frac{1}{\frac{4S_{r}^{2}}{Q^{2}x^{2}}-1},$$

(20) $$S_{t}=S_{r}+x\frac{dS_{r}}{dx}$$

and

(21) $$\frac{u}{r}=x\frac{dS_{r}}{dx}+(1-\nu )S_{r}.$$

From Equations (17) and (21), the boundary conditions Equation (16a,b) can be transformed into

(22a,b) $$\frac{u}{r}=x\frac{dS_{r}}{dx}+(1-\nu )S_{r}=(1-\nu )S_{0}\;\mathrm{and}\;W=0\;\mathrm{at}\;x=1.$$

Eliminating dW/dx from Equations (18) and (19), we can obtain an equation which contains only $S_{r}$

(23) $$x^{2}\frac{d^{2}S_{r}}{dx^{2}}+3x\frac{dS_{r}}{dx}+\frac{Q^{2}x^{2}}{8S_{r}^{2}-2Q^{2}x^{2}}=0.$$

Equation (23) can be further written as

(24) $$8xS_{r}{}^{2}\frac{d^{2}S_{r}}{dx^{2}}+24S_{r}{}^{2}\frac{dS_{r}}{dx}-2Q^{2}x^{3}\frac{d^{2}S_{r}}{dx^{2}}-6Q^{2}x^{2}\frac{dS_{r}}{dx}+Q^{2}x=0.$$

Expanding $S_{r}$ into the power series of the x, i.e., let

(25) $$S_{r}(x)=\sum_{n=0}^{\infty }b_{n}x^{n}.$$

Substituting Equation (25) into Equation (24) and substituting b for $b_{0}$, the general solution of Equation (24) can be written as

(26) $$S_{r}(x)=bf(\frac{Q^{2}x^{2}}{b^{3}}),$$

where b is an undetermined constant, and the function f(x) is shown in Appendix A. Furthermore, from Equations (18) and (26), one has

(27) $${\left(\frac{dW}{dx}\right)}^{2}=-2x^{2}\frac{d^{2}S_{r}}{dx^{2}}-6x\frac{dS_{r}}{dx}=-4x^{2}\frac{Q^{2}}{b^{2}}f^{\prime }(\frac{Q^{2}x^{2}}{b^{3}})-8x^{4}\frac{Q^{4}}{b^{5}}f^{\prime \prime }(\frac{Q^{2}x^{2}}{b^{3}})-12x^{2}\frac{Q^{2}}{b^{3}}f^{\prime }(\frac{Q^{2}x^{2}}{b^{3}}).$$

Then, Equation (27) yields

(28) $$\frac{dW}{dx}=-\frac{1}{2}\frac{Qx}{b}h(\frac{Q^{2}x^{2}}{b^{3}}),$$

where the function h(x) is shown in Appendix A. Further, we obtain from the integration of Equation (28)

(29) $$W=-\frac{1}{4}\frac{Qx^{2}}{b}g(\frac{Q^{2}x^{2}}{b^{3}})+A,$$

where A is another undetermined constant, and the function g(x) is shown in Appendix A.

The undetermined constants b and A can be determined by using the boundary conditions as follows. From Equations (22b) and (29), one has

(30) $$A=\frac{1}{4}\frac{Q}{b}g(\frac{Q^{2}}{b^{3}}).$$

Substituting (26) into (21),

(31) $$\frac{u}{r}=x\frac{dS_{r}}{dx}+(1-\nu )S_{r}=2\frac{Q^{2}}{b^{2}}x^{2}f^{\prime }(\frac{Q^{2}x^{2}}{b^{3}})+b(1-\nu )f(\frac{Q^{2}x^{2}}{b^{3}}).$$

From Equation (31), Equation (22a) gives

(32) $$2Q^{2}f^{\prime }(\frac{Q^{2}}{b^{3}})+b^{3}(1-\nu )f(\frac{Q^{2}}{b^{3}})=b^{2}(1-\nu )S_{0}.$$

Furthermore, from Equation (17a,b,c,d,e,f), Equation (32) can finally be transformed into

(33) $$2{\left(\frac{aq}{hE}\right)}^{2}f^{\prime }(\frac{a^{2}q^{2}}{h^{2}E^{2}b^{3}})+b^{3}(1-\nu )f(\frac{a^{2}q^{2}}{h^{2}E^{2}b^{3}})=b^{2}(1-\nu )\frac{\sigma_{0}}{E}.$$

Hence, for the given problem where a, h, E, ν, q and $\sigma_{0}$ are known in advance, the undetermined constant b can be determined by using Equation (33), and with the known constant b the undetermined constant A can also be determined by using Equation (30). So, all the undetermined constants can thus be determined.

Finally, from Equations (17) and (30), Equation (29) can be transformed into

(34) $$w(r)=-\frac{1}{4}\frac{a^{2}q}{bhE}\left[{\left(\frac{r}{a}\right)}^{2}g(\frac{q^{2}r^{2}}{h^{2}E^{2}b^{3}})-g(\frac{a^{2}q^{2}}{h^{2}E^{2}b^{3}})\right].$$

The maximum deflection of the membrane should be at the central point (r=0), that is

(35) $$w_{m}=w(0)=\frac{1}{4}\frac{a^{2}q}{bhE}g(\frac{a^{2}q^{2}}{h^{2}E^{2}b^{3}}).$$

Thus, the problem of axisymmetric deformation of the peripherally fixed circular membrane with initial stress under uniformly distributed transverse loads is analytically dealt with.

## 3. Synchronous Characterization Method of Surface and Interfacial Mechanical Properties of Thin-Film/Substrate Systems

**Figure 3:**
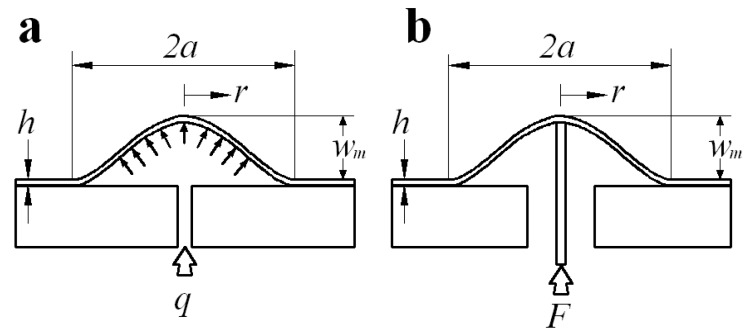
Schematics of the loading configuration of (**a**) a pressurized circular blister and (**b**) a shaft-loaded circular blister.

Dannenberg [38] first suggested using the blister test technique and subsequent investigators [39,40,41,42] developed this technique into many variant forms. In all blister tests, the driving force of delamination of thin-film/substrate is applied through a hole in the substrate (which reaches the interface of thin-film/substrate by boring or chemically etching). So, the blister tests are usually classified into two major variants: (i) fluid pressure loading (corresponding to a pressure blister test, as shown in Figure 3a), and (ii) shaft-loading (corresponding to a shaft-loaded blister test, as shown in Figure 3b). In the pressure blister test, the overhanging thin film is pressurized progressively by either a liquid or working gas (while in the shaft-loaded blister test by a shaft), until an axisymmetric blister crack runs into the interface of film–substrate. The interfacial energy of adhesion is then determined by measuring the debonding radius a, the blister height $w_{m}$, and the corresponding driving force q (or F). The axisymmetric blister geometry and the small angle at the crack front are the main attractions of this technique, in comparison with peeling tests. So, we here use the pressure blister test technique.

**Figure 4:**
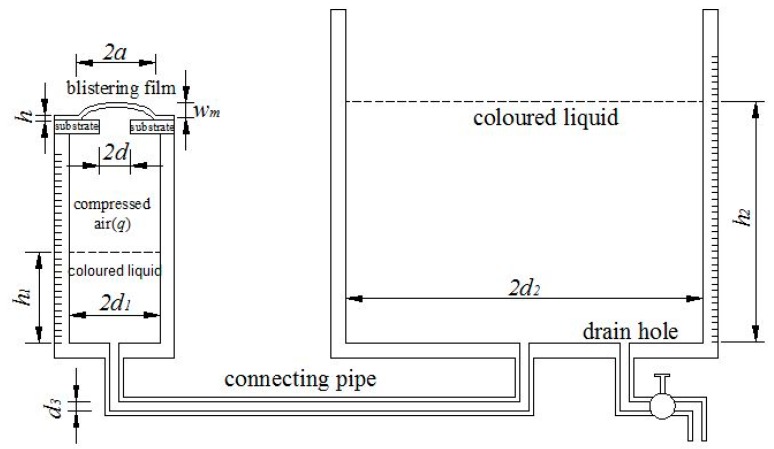
Schematic of testing setup for pressure blister test.

One disadvantage of the pressure blister test is that the strain energy release rate increases with the increase of blister radius, which may give rise to uncontrolled catastrophic debonding [43,44]. Moreover, the pressure blister test usually requires a sophisticated experimental setup to monitor simultaneously the changes in pressure and blister dimension, which may suffer from some problems such as possible dissolved gases and the soft compliance of the gaseous medium [42]. In order to overcome these disadvantages, in our earlier study we developed a novel loading method of pressure blister test [24], as illustrated in Figure 4. The two circular Plexiglas containers prepared both have a graduated scale in millimeter, and their wall thickness is about 10 mm, their height and inner radius (denoted by $h_{1}$, $h_{2}$, $d_{1}$, $d_{2}$) are 100 mm, 1500–2000 mm, 10–20 mm, and 100–400 mm (see Figure 4). A connecting pipe of inner radius 1 mm (denoted by $d_{3}$) is used to connect the two Plexiglas containers. The sample of thin-film/substrate systems is prepared particularly as follows. In the substrate of the specimen, a hole of radius d (about 1 mm), drilled or chemically etched, can reach the thin-film/substrate interface. Then, the substrate of the specimen needs to be tightly adhered onto the upper side of the smaller container (see Figure 4), so that such an adhesion can play a role in preventing air leakage. A colored liquid (the color plays only a role of eye-catching) is slowly poured into the bigger container and then flow into the smaller container via the connecting pipe. Such a pouring of colored liquid will give rise to the air enclosed in the smaller container to be compressed, resulting in a pressure loads q (compressed air) acting on the surface thin film of the sample via the hole in the substrate of the specimen. The interval time of each drop of the poured colored liquid is controlled about 1 min. The surface thin film under pressure loads q will be deformed slowly into a blister. The operation of pouring colored liquid is stopped until the blister obtained becomes measurable in size (the radius a is about 10–15 mm). The blister obtained needs some time to complete the deformation (no change in size), and then one can measure and record the radius a and height $w_{m}$ of the unchangeable (stable) blister. After this, the colored liquid poured needs to be discharged from the bigger container via the drain hole (i.e., unloading, see Figure 4), and the thickness h of the blistering thin film also needs to be measured and recorded.

Exactly, this static problem of equilibrium of the stable blistering film (no change in size) with initial stress (equal to the residual stress in the surface thin film of the specimen) is the large deflection problem of axisymmetrical deformation of a peripherally fixed circular membrane (radius a) with initial stress under the action of uniformly distributed transverse loads q, see Section 2. This problem has been solved and its closed-form solution has been presented (see Section 2). So, with the help of the closed-form solution presented in Section 2, we can further try to develop a synchronous characterization method of surface and interfacial mechanical properties of thin-film/substrate systems with residual stress. The relevant mechanical quantities need to be synchronously characterized include Young’s modulus of elasticity E, Poisson’s ratio ν, and residual stress $\sigma_{0}$ of surface thin films, the elastic strain energy stored in the blistering film (denoted by $U_{\mathrm{ef}}$), and the interfacial adhesion energy released per unit delamination area of thin-film/substrate (the energy release rate G). By studying the static problem of equilibrium of the stable blistering thin film, the surface mechanical properties E, ν, $\sigma_{0}$, and $U_{\mathrm{ef}}$ can be determined, while by studying the energy problem of equilibrium of forming this stable blistering thin film, the interfacial mechanical properties G can be determined. The particular way is detailed as follows.

### 3.1. Determination of Poisson’s Ratios, Young’s Modulus, and Residual Stress

The uniformly distributed transverse loads q (caused by the compressed air, see Figure 4) is applied to the surface thin film of a thin-film/substrate specimen via the hole of radius d, until it reaches $q^{\prime }$ and a stable blister (no change in size) with radius $a^{\prime }$ and height $w_{m}^{\prime }$ is observed. The applied loads $q^{\prime }$, blister radius $a^{\prime }$, and deflections $w_{m}^{\prime }$ and $w_{a\prime /2}^{\prime }$ are the parameters needing to be measured and recorded, where $w_{m}^{\prime }$ and $w_{a\prime /2}^{\prime }$ denote the thin film deflection w at r=0 and $r=a^{\prime }/2$, respectively, and the applied loads $q^{\prime }$ can be determined by studying the static problem of equilibrium after the colored liquid is poured into the bigger container (the detail derivation is shown in Section 3.2). After $q^{\prime }$, the applied loads q needs to be continued until it reaches $q^{\prime \prime }$ and another stable blister with radius $a^{\prime \prime }$ and height $w_{m}^{\prime \prime }$ is observed. The loads $q^{\prime \prime }$, blister radius $a^{\prime \prime }$, and deflections $w_{m}^{\prime \prime }$ and $w_{a\prime \prime /2}^{\prime \prime }$ also need to be measured and recorded. After unloading, the thickness h of the blistering thin film also needs to be measured and recorded. Suppose that the blistering thin film is kept always in elastic deformation during the loading. Then, with the measured and recorded parameters and with the help of the closed-form solution presented in Section 2, Poisson’s ratios ν, Young’s modulus E, and residual stress $\sigma_{0}$ are determined as follows.

When $q=q^{\prime }$, Equation (34) gives

(36) $$w_{m}^{\prime }=\frac{1}{4}\frac{{\left(a^{\prime }\right)}^{2}q^{\prime }}{b^{\prime }hE}g(\frac{{\left(a^{\prime }q^{\prime }\right)}^{2}}{h^{2}E^{2}{\left(b^{\prime }\right)}^{3}})$$

and

(37) $$w_{a\prime /2}^{\prime }=-\frac{1}{4}\frac{{\left(a^{\prime }\right)}^{2}q^{\prime }}{b^{\prime }hE}\left[\frac{1}{4}g(\frac{{\left(a^{\prime }q^{\prime }\right)}^{2}}{4h^{2}E^{2}{\left(b^{\prime }\right)}^{3}})-g(\frac{{\left(a^{\prime }q^{\prime }\right)}^{2}}{h^{2}E^{2}{\left(b^{\prime }\right)}^{3}})\right].$$

Hence, the undetermined constant $b^{\prime }$ and Young’s modulus E can be determined by using Equations (36) and (37). Then, with the known $b^{\prime }$ and E, Equation (33) gives

(38) $$4{\left(\frac{a^{\prime }q^{\prime }}{hE}\right)}^{2}f^{\prime }(\frac{{\left(a^{\prime }q^{\prime }\right)}^{2}}{h^{2}E^{2}{\left(b^{\prime }\right)}^{3}})+2{\left(b^{\prime }\right)}^{3}(1-\nu )f(\frac{{\left(a^{\prime }q^{\prime }\right)}^{2}}{h^{2}E^{2}{\left(b^{\prime }\right)}^{3}})=\frac{2\sigma_{0}}{E^{1/3}}{\left(b^{\prime }\right)}^{2}(1-\nu ).$$

Note that Equation (38) contains only two undetermined parameters, Poisson’s ratios ν and residual stress $\sigma_{0}$.

While $q=q^{\prime \prime }$, Equation (34) gives

(39) $$w_{m}^{\prime \prime }=\frac{1}{4}\frac{{\left(a^{\prime \prime }\right)}^{2}q^{\prime }}{b^{\prime \prime }hE}g(\frac{{\left(a^{\prime \prime }q^{\prime \prime }\right)}^{2}}{h^{2}E^{2}{\left(b^{\prime \prime }\right)}^{3}})$$

and

(40) $$w_{a\prime \prime /2}^{\prime \prime }=-\frac{1}{4}\frac{{\left(a^{\prime \prime }\right)}^{2}q^{\prime \prime }}{b^{\prime \prime }hE}\left[\frac{1}{4}g(\frac{{\left(a^{\prime \prime }q^{\prime \prime }\right)}^{2}}{4h^{2}E^{2}{\left(b^{\prime \prime }\right)}^{3}})-g(\frac{{\left(a^{\prime \prime }q^{\prime \prime }\right)}^{2}}{h^{2}E^{2}{\left(b^{\prime \prime }\right)}^{3}})\right].$$

Hence, the undetermined constant $b^{\prime \prime }$ and Young’s modulus E can also be determined by using Equations (39) and (40). If the value of Young’s modulus E (obtained in this time) is very close to the value obtained in $q=q^{\prime }$, then this indicates that the blistering thin film is kept always in elastic deformation during the loading, otherwise, the test results are invalid (prepare another thin-film/substrate specimen again for test). If the results are valid (there may be some error caused usually by measurement), then, with the known $b^{\prime \prime }$ and E, Equation (33) gives

(41) $$4{\left(\frac{a^{\prime \prime }q^{\prime \prime }}{hE}\right)}^{2}f^{\prime }(\frac{{\left(a^{\prime \prime }q^{\prime \prime }\right)}^{2}}{h^{2}E^{2}{\left(b^{\prime \prime }\right)}^{3}})+2{\left(b^{\prime \prime }\right)}^{3}(1-\nu )f(\frac{{\left(a^{\prime \prime }q^{\prime \prime }\right)}^{2}}{h^{2}E^{2}{\left(b^{\prime \prime }\right)}^{3}})=\frac{2\sigma_{0}}{E^{1/3}}{\left(b^{\prime \prime }\right)}^{2}(1-\nu ).$$

So, Poisson’s ratios ν and residual stress $\sigma_{0}$ can thus be determined by using Equations (38) and (41).

### 3.2. Determination of Elastic Strain Energy $U_{ef}$ Stored in Blistering Thin Films

The volume under the blistering thin film is

(42) $$V=\pi a^{2}\frac{1}{a}\int_{0}^{a}w(r)dr=\pi a\int_{0}^{a}w(r)dr.$$

Studying the static problem of equilibrium of the loading system, one has

(43) $$q\pi d_{1}^{2}=\rho g(h_{2}-h_{1})\pi d_{2}^{2},$$

where ρ is the density of the liquid and g is the acceleration of gravity. Further, Equation (43) gives

(44) $$q=\rho g{\left(d_{2}/d_{1}\right)}^{2}(h_{2}-h_{1}).$$

Thus, the elastic strain energy stored in the blistering thin film can finally be written as

(45) $$U_{\mathrm{ef}}=Vq=\pi a\rho g{\left(d_{2}/d_{1}\right)}^{2}(h_{2}-h_{1})\int_{0}^{a}w(r)dr.$$

Obviously, here $U_{\mathrm{ef}}$ should be a function of the blister radius a.

### 3.3. Determination of Work $U_{F}$ Caused by Liquid Potential Energy Change

It can easily be proved that the gravitational potential energy of the liquid of volume $\pi d^{2}h$ can be expressed in $\pi \rho gd^{2}h^{2}/2$, where d is the radius of a circular container, h is the height of the liquid in the circular container. So, as shown in Figure 4, the total change of the gravitational potential energy of the poured colored liquid in two containers, i.e., the work done by the poured colored liquid as external force to the loading system, is

(46) $$U_{F}=\frac{1}{2}\pi \rho gd_{2}{}^{2}{\left(h_{2}+\frac{d_{1}{}^{2}}{d_{2}{}^{2}}h_{1}\right)}^{2}-\frac{1}{2}\pi \rho gd_{2}{}^{2}h_{2}{}^{2}-\frac{1}{2}\pi \rho gd_{1}{}^{2}h_{1}{}^{2}.$$

Equation (46) can further be simplified into

(47) $$U_{F}=\frac{1}{2}\pi \rho gd_{1}{}^{2}h_{1}\left(2h_{2}-\frac{d_{2}{}^{2}-d_{1}{}^{2}}{d_{2}{}^{2}}h_{1}\right).$$

### 3.4. Determination of Elastic Strain Energy $U_{\mathrm{ea}}$ Stored in Enclosed Compressed Air

The elastic strain energy $U_{\mathrm{ea}}$ stored in the compressed air enclosed in the container of radius $d_{1}$ can be determined via a graph of the function $U_{\mathrm{ea}}(q)$ or $U_{\mathrm{ea}}(h_{2}-h_{1})$, which can be obtained via an “assisted synchronous experiment”. The so-called “assisted synchronous experiment” is detailed as follows. Before a concrete pressure blister test, a flat steel plate is put onto the upper surface of the specimen (over the hole of radius d) to prevent the deformation of thin film during loading. Then, the colored liquid is, step by step, poured into the container of radius $d_{2}$, and in each step the amount of the colored liquid poured needs to keep consistent as much as possible. By studying the static problem of equilibrium in each step, one has

(48) $$q_{i}\cdot \pi d_{1}^{2}=\rho g(h_{2i}-h_{1i})\pi d_{2}^{2}.$$

Hence,

(49) $$q_{i}=\rho g{\left(d_{2}/d_{1}\right)}^{2}(h_{2i}-h_{1i}),$$

where, $h_{1i}$ and $h_{2i}$ denote the liquid height in the containers of radius $d_{1}$ and $d_{2}$ after the operation of step i, and $q_{i}$ denotes the pressure caused by the compressed air enclosed in the container of radius $d_{1}$ after the operation of step i. The work done by the external force to the system, caused by the change in the amount of the colored liquid poured after the operation of step i, can be written as, from Equation (47),

(50) $${U_{F}|}_{i}=\frac{1}{2}\pi \rho gd_{1}^{2}h_{1i}(2h_{2i}-\frac{d_{2}^{2}-d_{1}^{2}}{d_{2}^{2}}h_{1i}).$$

According to the law of conversation of energy, the elastic strain energy stored in the compressed air enclosed in the container of radius $d_{1}$ after the operation of step i, ${U_{\mathrm{ea}}|}_{i}$, should equal to ${U_{F}|}_{i}$, that is,

(51) $${U_{\mathrm{ea}}|}_{i}=\frac{1}{2}\pi \rho gd_{1}^{2}h_{1i}(2h_{2i}-\frac{d_{2}^{2}-d_{1}^{2}}{d_{2}^{2}}h_{1i}).$$

Step by step, ${U_{\mathrm{ea}}|}_{i}$ and $q_{i}$ or $\left(h_{2i}-h_{1i}\right)$ can be recorded down, hence a graph of the function $U_{\mathrm{ea}}(q)$ or $U_{\mathrm{ea}}(h_{2}-h_{1})$ can finally be plotted on a coordinates plane. So, in the pressure blister test that followed, using the obtained graph of the function $U_{\mathrm{ea}}(q)$ or $U_{\mathrm{ea}}(h_{2}-h_{1})$, the elastic strain energy $U_{\mathrm{ea}}$ stored in the compressed air enclosed in the container of radius $d_{1}$ can thus be determined, because the volume of the air enclosed originally in the container of radius $d_{1}$ in the “assisted synchronous experimentation” is equal to that in the following pressure blister test.

### 3.5. Determination of the Energy Release Rate G

In the light of the law of conversation of energy, the work $U_{F}$ caused by the change in the amount of the colored liquid poured minus the elastic strain energy $U_{\mathrm{ef}}$ stored in the stable blistering film and the elastic strain energy $U_{\mathrm{ea}}$ caused by the air compression equals exactly to the total energy released during the film–substrate delamination from the radius d to the radius a. So, for the thin films adhered uniformly to a rigid substrate, the interfacial adhesion energy released per unit delamination area of thin-film–substrate, i.e., the so-called energy release rate, can finally be written as

(52) $$G=\frac{d}{dS}(U_{F}-U_{\mathrm{ef}}-U_{\mathrm{ea}})=\frac{1}{\pi (a^{2}-d^{2})}(U_{F}-U_{\mathrm{ef}}-U_{\mathrm{ea}}).$$

## 4. Results and Discussion

A numerical example of a circular rubber thin film with a = 20 mm, h = 1 mm, E = 7.84 MPa, ν = 0.47, $\sigma_{0}$ = 3 MPa subjected to the uniformly distributed transverse loads q = 0.5 MPa is considered, to discuss the influence of the initial stress and small-rotation-angle assumption of membrane on the solution to circular problems. We here use four solutions to conduct the numerical calculation: the extended Hencky solution presented in [35] (considering initial stress but obeying small-rotation-angle assumption of membrane), the solution presented in this study (considering initial stress and giving up small-rotation-angle assumption, see Section 2), the well-known Hencky solution presented in [30] (considering no initial stress and obeying small-rotation-angle assumption), and the solution presented in [36] (giving up small-rotation-angle assumption but considering no initial stress).

**Figure 5:**
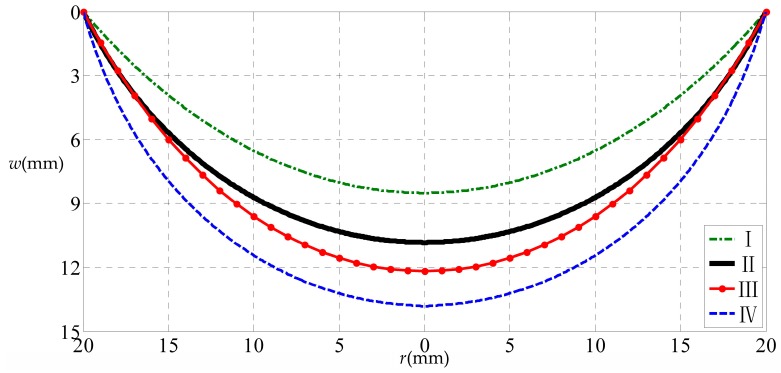
Variation of w with r. Where, I denotes considering initial stress but obeying small-rotation-angle assumption of membrane; II denotes considering initial stress and giving up small-rotation-angle assumption; III denotes considering no initial stress and obeying small-rotation-angle assumption; IV denotes giving up small-rotation-angle assumption but considering no initial stress.

Figure 5 shows the numerical calculation results of the variation of w with r, where the uppermost dash–dot line represents the result obtained by using the solution presented in [35], the downward second one (the heavy solid line) by using the solution presented in this study, the downward third one (the solid line with small dots ) by the well-known Hencky solution [30], and the down most dashed line by the solution presented in [36]. From Figure 5 it can be seen that, the difference between the various situations is obvious, where the difference between the uppermost line and the downward second one is caused by only the small-rotation-angle assumption of membrane, the difference between the down most line and the downward second one is caused by only the initial stress, and the difference between the downward third line and the downward second one is caused by both the initial stress and the small-rotation-angle assumption of membrane. So, in light of this, the residual stress in the surface thin film of thin-film/substrate systems (corresponding to the initial stress in circular membrane problems) have a large influence on the synchronous characterization, while at the same time, the small-rotation-angle assumption of membrane should be given up in order to improve the accuracy of theoretical solution.

**Figure 6:**
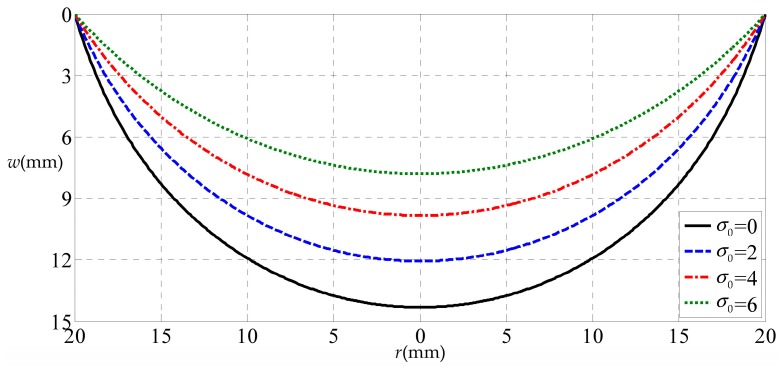
Variation of w with r when $\sigma_{0}$ take different values.

Figure 6 shows the deflection differences of the circular rubber thin film with different initial stresses ($\sigma_{0}$ = 0, 2, 4 and 6 MPa), i.e., the effect of residual stress on the deflection of a blistering thin film. Figure 6 indicates that the deflection will decrease along with the increase of initial stress (corresponding to the residual stress of thin-film/substrate systems). This should be easily understood, because the initial stress (or residual stress) will give rise to the increase of transverse stiffness of the membrane, resulting in the decrease of deflection of the membrane.

In our earlier study [5], we use the shaft-loaded blister test technique for synchronous characterization, and the theoretical solution presented in [5] is still a one with the small-rotation-angle assumption of membrane, except considering no initial stress in membranes. In fact, the shaft loading is very easily to give rise to the stress concentration, while the pressure loading can overcome the disadvantage of stress concentration. In this sense, the pressure blister test ought to be the preferred technique.

It should be pointed out that, for the thin-film/substrate systems by polymer adhesives, the polymer adhesive will adhere to the surface thin film or the substrate, after the delamination of thin-film/substrate. So, if it adheres to the surface thin film, then the determined surface mechanical properties (Poisson’s ratios and Young’s modulus of elasticity, including residual stress and elastic strain energy stored in blistering thin films) is unavoidably affected by the polymer adhesive on the surface thin film. We mean that, in this situation, the determined Poisson’s ratios and Young’s modulus of elasticity cannot be understood as the inherent properties of the thin film characterized. From Section 3, it can be seen that, however, the determined interfacial adhesion energy released per unit delamination area of thin-film/substrate (the energy release rate) will not be affected by this situation.

## 5. Conclusions

In this study, based on the novel loading method of pressure blister test developed in our earlier study, a theoretical study on the synchronous characterization technique of surface and interfacial mechanical properties of thin-film/substrate systems with residual stress was presented. In comparison with the existing works, the innovation of this study is mainly reflected in the following two aspects: a new closed-form solution, taking the residual stress in surface thin films into account and giving up the small-rotation-angle assumption of membrane, was presented (see Section 2); based on the presented closed-form solution, the synchronous characterization technique taking residual stress into account is given for the first time (see Section 3). The inadequacy of this study is that the synchronous characterization technique presented here is still at the theoretical stage and failed to carry out experimental research (due to the lack of high-precision deflection measuring instrument at this stage).

In conclusion, this study ought to have significant implication in thin-film mechanics and mechanical characterization of thin-film/substrate systems, but more theoretical and experimental research still need to be conducted.

## Appendix A

$$\begin{array}{ll} f(x) & =1-\frac{1}{2^{6}}x-\frac{1}{3\;\times \;2^{11}}(1+8b)x^{2}-\frac{1}{9\;\times \;2^{19}}(13+224b+768b^{2})x^{3}\\ & -\frac{1}{45\;\times \;2^{25}}(85+2336b+17664b^{2}+36864b^{3})x^{4}-\frac{1}{675\;\times \;2^{30}}(925+35616b\\ & +433664b^{2}+2002944b^{3}+2949120b^{4})x^{5}-\frac{1}{14175\;\times \;2^{36}}(30125+1510752b\\ & +25919488b^{2}+193241088b^{3}+628162560b^{4}+707788800b^{5})x^{6}\\ & -\frac{1}{99225\;\times \;2^{46}}(5481025+341602560b+7663878656b^{2}+80670326784b^{3}\\ & +423116734464b^{4}+1048093655040b^{5}+951268147200b^{6})x^{7}-\ldots \ldots \end{array},$$

$$\begin{array}{ll} h(x) & =1+\frac{1}{2^{6}}(1+8b)x+\frac{1}{3\;\times \;2^{12}}(5+88b+288b^{2})x^{2}\\ & +\frac{1}{9\;\times \;2^{19}}(55+1568b+11712b^{2}+23040b^{3})x^{3}+\frac{1}{45\;\times \;2^{25}}\\ & \times (525+21176b+259264b^{2}+1165824b^{3}+1612800b^{4})x^{4}+\frac{1}{675\;\times \;2^{31}}\\ & \times (15375+813112b+14179808b^{2}+104706048b^{3}+329057280b^{4}\\ & +348364800b^{5})x^{5}+\frac{1}{14175\;\times \;2^{38}}(1278825+84455776b+1940012480b^{2}\\ & +20441522688b^{3}+105313941504b^{4}+251315896320b^{5}+214592716800b^{6})x^{6}\\ & +\frac{1}{99225\;\times \;2^{46}}(71612125+5709209280b+164336526592b^{2}+2284570114048b^{3}\\ & +16790465630208b^{4}+65487716352000b^{5}+125080613683200b^{6}\\ & +89270570188800b^{7})x^{7}+\ldots \ldots \end{array},$$

$$\begin{array}{ll} g(x) & =1+\frac{1}{2^{7}}(1+8b)x+\frac{1}{9\;\times \;2^{12}}(5+88b+288b^{2})x^{2}\\ & +\frac{1}{9\;\times \;2^{21}}(55+1568b+11712b^{2}+23040b^{3})x^{3}+\frac{1}{225\;\times \;2^{25}}\\ & \times (525+21176b+259264b^{2}+1165824b^{3}+1612800b^{4})x^{4}+\frac{1}{2025\;\times \;2^{32}}\\ & \times (15375+813112b+14179808b^{2}+104706048b^{3}+329057280b^{4}\\ & +348364800b^{5})x^{5}+\frac{1}{99225\;\times \;2^{38}}(1278825+84455776b+1940012480b^{2}\\ & +20441522688b^{3}+105313941504b^{4}+251315896320b^{5}+214592716800b^{6})x^{6}\\ & +\frac{1}{99225\;\times \;2^{49}}(71612125+5709209280b+164336526592b^{2}+2284570114048b^{3}\\ & +16790465630208b^{4}+65487716352000b^{5}+125080613683200b^{6}\\ & +89270570188800b^{7})x^{7}+\ldots \ldots \end{array}.$$

## References

[1] Kwon J.-H., Zhang X., Piao S.H., Choi H.J., Bae J.-H., Park J. (2017). Stability Study of Flexible 6,13-Bis(triisopropylsilylethynyl)pentacene Thin-Film Transistors with a Cross-Linked Poly(4-vinylphenol)/Yttrium Oxide Nanocomposite Gate Insulator. Polymers.

[2] Hatami J., Silva S.G., Oliveira M.B., Costa R.R., Reis R.L., Mano J.F. (2017). Multilayered Films Produced by Layer-by-Layer Assembly of Chitosan and Alginate as a Potential Platform for the Formation of Human Adipose-Derived Stem Cell aggregates. Polymers.

[3] Wang C.G., Kang J.T., Xue Z.M., Tan H.F. (2017). Buckling induced delamination and microflow analysis of film/substrate system. Compos. Struct..

[4] Schultrich B. (1985). Modified propagation of ultrasonic surface waves by thin elastic or viscous films. Z. Angew. Math. Mech..

[5] Sun J.Y., Lian Y.S., Li Z.L., He X.T., Zheng Z.L. (2014). Theoretical study on shaft-loaded blister test technique: Synchronous characterization of surface and interfacial mechanical properties. Int. J. Adhes. Adhes..

[6] Shojaei O.R., Kruml T., Karimi A., Martin J.L. (1998). Mechanical properties of TiN thin films investigated using biaxial tensile testing. Surf. Eng..

[7] Weihs T.P., Hong S., Bravman J.C., Nix W.D. (1988). Mechanical deflection of cantilever microbeams: A new technique for testing the mechanical properties of thin films. J. Mater. Res..

[8] Moses S., Witt R.K. (1949). Evaluation of adhesion by ultrasonic vibrations. Ind. Eng. Chem. Res..

[9] Shagaev V.V., Lin T.T. (2017). Ferrite films with enhanced stability of ferromagnetic resonance frequency. Tech. Phys..

[10] Liu S.B., Wang Q.J. (2007). Determination of Young’s modulus and Poisson’s ratio for coatings. Surf. Coat. Technol..

[11] Bamber M.J., Cooke K.E., Mann A.B., Derby B. (2001). Accurate determination of Young’s modulus and Poisson’s ratio of thin film by a combination of acoustic microscopy and nanoindentation. Thin Solid Films.

[12] Reichert W.M., Lves J.T., Suci P.A., Hlady V. (1987). Excitation of fluorescent emission from solutions at the surface of polymer thin-film waveguides: An integrated optics technique for the sensing of fluorescence at the polymer/solution interface. Appl. Spectrosc..

[13] Gao Y., Li L.Y., Tan P.H., Liu L.Q., Zhang Z. (2010). Application of Raman spectroscopy in carbon nanotube-based polymer composites. Chin. Sci. Bull..

[14] Sun J.Y., Hu J.L., Zheng Z.L., He X.T., Geng H.H. (2011). A practical method for simultaneous determination of Poisson’s ratio and Young’s modulus of elasticity of thin films. J. Mech. Sci. Technol..

[15] Sneddon I.N. (1948). Boussinesq’s problem for a rigid cone. Math. Proc. Camb..

[16] Kuwahara K., Nakagawa T., Kuramasu K. (1971). Effect of ion-pump evacuation on the adhesion of evaporated thin films. Metals. Trans..

[17] Beams J.W. (1954). Production and use of high centrifugal fields. Science.

[18] Faure R., Carlan A., Crebassa J., Desvousseaux G., Robrieux B. (1972). Modification de la structure des couches minus d’argent soumiser a des vibrations mechaniques measure del’adhesion. Thin Solid Films.

[19] Willians R.C., Backus R.C. (1949). The electron-micrographic structure of shadow-cast films and surfaces. J. Appl. Phys..

[20] Chen W.T., Flavin T.F. (1972). Mechanics of film adhesion: Elastic and elastic-plastic behavior. IBM J. Res. Dev..

[21] Lin D.S. (1971). The adhesion of metal films to glass and magnesium oxide in tangential shear. J. Appl. Phys..

[22] Belajamin P., Weaver C. (1960). Measurement of adhesion of thin films. Proc. R. Soc..

[23] Chang J.Y., Yu G.P., Huang J.H. (2009). Determination of Young’s modulus and Poisson’s ratio of thin films by combining sin2ψ X-ray diffraction and laser curvature methods. Thin Solid Films.

[24] Sun J.Y., Qian S.H., Li Y.M., He X.T., Zheng Z.L. (2013). Theoretical study of adhesion energy measurement for film/substrate interface using pressurized blister test: Energy release rate. Measurement.

[25] Jin C. (2008). Analysis of energy release rate and bending-to-stretching behavior in the shaft-loaded blister test. Int. J. Solids. Struct..

[26] Hencky H. (1915). Über den Spannungszustand in kreisrunden Platten mit verschwindender Biegungssteifigkeit. Zeitschrift Für Mathematik und Physik.

[27] Chien W.Z. (1948). Asymptotic behavior of a thin clamped circular plate under uniform normal pressure at very large deflection. Sci. Rep. Nat. Tsing Hua Univ..

[28] Alekseev S.A. (1953). Elastic circular membranes under the uniformly distributed loads. Eng. Corpus.

[29] Arthurs A.M., Clegg J. (1994). On the solution of a boundary value problem for the nonlinear Föppl-Hencky equation. Z. Angew. Math. Mech..

[30] Sun J.Y., Rong Y., He X.T., Gao X.W., Zheng Z.L. (2013). Power series solution of circular membrane under uniformly distributed loads: Investigation into Hencky transformation. Struct. Eng. Mech..

[31] Yang Z.X., Sun J.Y., Ran G.M., He X.T. (2017). A new solution to Föppl-Hencky membrane equation. J. Mech..

[32] Wauer J., Plaut R.H. (1991). Vibrations of an extensible, air-inflated, cylindrical membrane. Z. Angew. Math. Mech..

[33] Weinitschke H.J. (1973). Endliche deformationen elastischer membrane. Z. Angew. Math. Mech..

[34] Kolesnikov A.M., Zubov L.M. (2009). Large bending deformations of a cylindrical membrane with internal pressure. Z. Angew. Math. Mech..

[35] Sun J.Y., Lian Y.S., Li Y.M., He X.T., Zheng Z.L. (2015). Closed-form solution of elastic circular membrane with initial stress under uniformly-distributed loads: Extended Hencky solution. Z. Angew. Math. Mech..

[36] Lian Y.S., Sun J.Y., Yang Z.X., He X.T., Zheng Z.L. (2016). Closed-form solution of well-known Hencky problem without small-rotation-angle assumption. Z. Angew. Math. Mech..

[37] Wan K.T., Guo S., Dillard D.A. (2003). A theoretical and numerical study of a thin clamped circular film under an external load in the presence of a tensile residual stress. Thin Solid Films.

[38] Dannenberg H. (1961). Measurement of adhesion by a blister method. J. Appl. Polym. Sci..

[39] Malyshev B.M., Salganik R.L. (1965). The strength of adhesive joints using the theory of cracks. Int. J. Fract..

[40] Williams M.L. (1969). The continuum interpretation for fracture and adhesion. J. Appl. Polym. Sci..

[41] Bennett S.J., Devries K.L., Williams M.L. (1974). Adhesion fracture mechanics. Int. J. Fract..

[42] Wan K.T., Mai Y.W. (1995). Fracture mechanics of a shaft-loaded blister of thin flexible membrane on rigid substrate. Int. J. Fract..

[43] Lai Y.H., Dillard D.A. (1994). A study of the fracture efficiency parameter of blister tests for films and coatings. J. Adhes. Sci. Technol..

[44] Sun Z., Wan K.T., Dillard D.A. (2004). A theoretical and numerical study of thin film delamination using the pull-off test. Int. J. Solids Struct..

